# One's Interoception Affects the Representation of Seeing Others' Pain: A Randomized Controlled qEEG Study

**DOI:** 10.1155/2021/5585060

**Published:** 2021-04-03

**Authors:** Michela Balconi, Laura Angioletti

**Affiliations:** ^1^International Research Center for Cognitive Applied Neuroscience (IrcCAN), Catholic University of the Sacred Heart, Milan, Italy; ^2^Research Unit in Affective and Social Neuroscience, Department of Psychology, Catholic University of the Sacred Heart, Milan, Italy

## Abstract

**Objective:**

This research demonstrates that interoceptive attentiveness (IA) can modulate cortical oscillations related to the emotional and cognitive representations of observing pain in others.

**Methods:**

Twenty participants were required to observe painful/nonpainful stimuli in an individual versus the interactive condition during the recording of the electroencephalogram. The sample was divided into experimental (EXP) and control (CTR) groups, and the EXP group was explicitly required to direct the attention on its interoceptive correlates while observing the stimuli.

**Results:**

Mixed repeated measures, analyses of variance, were applied to each EEG frequency band. Significant findings were obtained mainly for theta and beta bands for the two groups. A hemispheric lateralisation effect was found, with right lateralisation of the theta band for the EXP group when observing painful stimuli and enhanced left activation of theta and beta bands for the CTR group when observing nonpainful stimuli. For both groups, frontal cortical regions were significantly sensitive to social scenarios, while posterior parietal activation was found for stimuli depicting the individual condition.

**Conclusions:**

The results suggest that IA might enhance the emotional representation of painful stimuli, highlighting their negative and unpleasant features in the EXP group, while the attention of the CTR group was mainly drawn to nonpainful stimuli in social and individual conditions, with a positive valence. The role of frontal regions in the processing of social stimuli through social cognition, inducing emotional mirroring and requiring deeper analysis of the social context, was underlined. We propose that IA could be trained for promoting emotion regulation and empathic response.

## 1. Introduction

Conventionally, interoception has been defined as the perception of the afferent signals on the body state and homeostasis, mainly relegating it to the physiological condition of the body. However, a recent broader definition of the construct combined anatomy and motivational levels and included neural correlates and mental representation of the internal changes of the body (i.e., the cortical signals of the relation between basic bodily perception and its effects on higher-order cognitive processing) [1, 2]. In this sense, interoception can be conceived as a type of perception that starts from physiology as a bodily experience that is related to homeostasis, which, however, encompasses neural correlates (e.g., insular cortex activity) supporting mentalized representational states of the physiological condition of the body [3]. These representational states have different degrees of awareness, ranging from unconscious to conscious levels [3]. Also, the representation of these bodily responses informs different states of subjective experience that may contribute to emotion-specific feelings [2]. Accordingly, it is possible to consider the complex interplay between mind and body as a dynamic “interoceptive experience.”

From James and Lange theory [4] to predictive coding theories [5], it has been suggested that internal physiological sensations (and the representations of these somatic processes [6]) can evoke emotional responses and affective states in individuals (and vice versa), accordingly influencing their behavioural response. Within this context, interoception is configured as the way through which the sensory and cognitive processes that are connected to emotions are perceived as changes in body conditions and are encoded in affective feelings [7, 8]. The most advanced cortical portions of the neural system (prefrontal cortex [PFC], dorsolateral prefrontal cortex [DLPFC], ventromedial PFC, and insular cortex) predict, confirm, and update these affective feelings and orchestrate the behaviour of the person with themselves, with others, and within the complex systems [7]. However, despite previous studies suggesting a matrix of neural regions that support interoception [6, 9, 10], not all cortical correlates involved in interoceptive representations.

Moreover, interoception has been conceived as a relatively stable trait; however, it may also vary according to the modulation of specific variables, such as the degree to which a person focuses the attention on bodily changes [11, 12], either self-awareness [13] or emotional experiences [7]. The focused attention on a particular interoceptive signal (e.g., heart rate or breath) for a given time interval is known as interoceptive attentiveness (IA), and it has not been exhaustively determined so far [10].

In this study, a different way was proposed to explore if and how the direct attention to one's interoceptive changes (the variations of the internal state) may influence the neurophysiological correlates related to the mental representation (both cognitive and emotional) of observing painful scenarios. Participants were asked to focus their attention on their bodily and mental modifications while observing a highly salient experience, that is, a painful scenario. The decision to explore the dimension of the observation of pain in others from an interoceptive perspective is based on at least three key reasons: (i) the highly connoted emotional characteristics of the observation of pain in others; (ii) the marked neurophysiological and psychophysiological activation that is traceable following the exposure to a stimulus, evoking empathy for pain [14–19]; and (iii) the involvement of strictly sensorial and affective cortical areas. Indeed, since the observation of pain in others has been shown to activate neural systems, processing the sensory, affective, and cognitive representational components of pain [20, 21]; it can be a useful framework to deepen the effect of IA modulation on these multiple representational levels.

Previously, several studies focused on the emotional and sensory activations derived from passively observing the painful state of others [21, 22], and this line of studies gradually began to differentiate from research that investigates the physical aspects of direct painful experience [23, 24]. The observation of pain in others includes the activation of mirror neural systems, which render the understanding of a painful situation possible because such observation activates similar neural circuits as if we were experiencing the sensations and emotions of that pain ourselves [25]. This phenomenon, known as the “vicarious experience” of pain, has been previously explained by the perception-action model of empathy as an expected perception of the state of the object that is receiving painful stimulation, which automatically activates the observer's representation of the situation and the state of the object observed. The activation of these representations automatically triggers and generates the associated autonomous and somatic responses in the observer [26]. However, the observation of pain in others does not necessarily activate the representations of somatic pain since it is mainly a reflexive and cognitive experience in which empathy plays a fundamental role in activating emotional and cognitive representations of the situation [23, 27].

Several neuroimaging studies have explored the neural processes and cortical correlates underneath the observation of pain in others [21, 22, 24]. The involvement of primary and secondary somatosensory cortices and motor regions during the observation of other people's pain has been demonstrated by an extensive body of research [28], while empathising with the pain of others seems to recruit the whole pain matrix, including a set of frontal regions, such as cingulate cortices and the PFC, which are particularly involved in the affective and motivational evaluation of pain and the cognitive attentional dimension of pain, respectively [29–31]. Moreover, brain evidence suggests that the observation of others' pain may rely on the activation of regions that are not normally linked to pain perception but more to social cognition processes, such as interoception, emotional learning, and social-cue processing [25]. For instance, frontal, premotor, parietal, and amygdala regions [16] and left lateral occipital cortex activations [18] were found during the observation of pain in others, as well as fronto-parietal network activity, that is specialised for representing one's own and others' responses to painful stimuli [23, 24].

By connecting interoception to the observation of pain experienced by another individual, it has been shown that when visual stimuli describing other people in painful conditions are observed, higher interoceptive sensitivity (IS) (determined by a heartbeat perception task) can be associated with a greater estimated degree of pain (result interpreted as representative of cognitive empathy) as well as a greater state of arousal and feelings of compassion (evidence indicating the activation of the affective component of empathy) [32]. Sharing the activation of these circuits at a neurophysiological level also allows individuals to activate their body's representations of pain when they observe someone who suffers, leading to more intense empathic responses [33, 34]. Despite previous studies conceiving interoception dimensions (i.e., IS and interoceptive awareness) as relatively stable traits that can modulate both the subjective experience of emotion and the ability of subjects to distinguish “self” from “others” in the empathic resonance of an action [35], other research studies have shown that cortical representations of interoceptive attention can be modulated by specific training [12]. Indeed, according to Bowling and colleagues [36], conscious vicarious pain perception could also be enhanced by an attentional focus on the internal emotional and physical states in typical adults; albeit, the authors did not test this hypothesis at the neurophysiological level.

Specifically, relatively few studies have investigated the link between the observation of pain in others and interoceptive representations by exploring their effect on cortical oscillations [14]. Cortical oscillations are involved in many perceptual and cognitive operations [37–39], and their systematic assessment might provide a unique window into the processes that underly the vicarious experience of pain [40], the maturation of empathy for pain, which is a process that is supported by interoception [14] and interoceptive representations themselves.

Previously, frequency band analysis proved the involvement of the sensorimotor system in the observation of action, touch, and pain [19, 41]. Particularly, the observation of pain in others was shown to suppress somatosensory cortex oscillations [42], with alpha and beta bands suppressed at both posterior and central sensors for both pain and no-pain conditions [19]. In a recent study involving motor cortical suppression, alpha and beta bands were shown to underly the sensory qualities of others' pain, while the gamma band was suggested to reflect the cognitive aspects [43]. Moreover, mu and beta suppressions were only found to be significant electrophysiological markers in the sensory and localised pain responders, compared to affective nonlocalised responders and controls [41]. Increased theta and decreased alpha band effects were also found for the observation of painful stimuli [15]. Specifically, the theta band was positively correlated with subjective ratings of perceived pain and self-unpleasantness, suggesting that theta oscillations are involved in emotional sharing during empathy for pain [15]. Theta rhythm modulation was also associated with affective valence discrimination of visual displays [44–50], activation of memory, and emotion regulation systems [51].

This study seeks to fill the following gaps in the literature: (i) the knowledge of cortical correlates involved in interoceptive representations; (ii) the effects of attentional focus on the internal emotional and physical states in typical adults at the neurophysiological level; (iii) the effect that IA modulation may have on brain cortical oscillations (in terms of frequency bands) related to emotional, cognitive, and sensory levels in empathy for pain. Specifically, the present study aims to answer two main questions: which cortical effect does the focus on one's interoceptive feelings have on the subjective experience of observing the pain experienced by another individual? Could greater IA modulate the affective and cognitive empathic response at the neurophysiological level while observing pain in others?

For this reason, the purpose of this study was to test if a focus on one's interoceptive states may affect the mental representation of the other painful states. Specifically, we aimed to explore cortical oscillations related to the modulation of IA during the observation of painful vs nonpainful stimuli presented in a social or individual scenario. We hypothesized that the cortical oscillations related to emotional, cognitive, and sensory representations of the observation of pain in others can be enhanced by the modulation of IA in the experimental (EXP) group, which was explicitly required to focus their attention on bodily sensations and cognitive and emotional correlates while observing a set of painful/nonpainful stimuli, compared to the control (CTR) group, which was instructed to observe the stimuli only. The null hypothesis will be that IA modulation has no effect on cortical oscillations in the EXP, compared to the CTR group.

## 2. Methods

### 2.1. Sample

A total of 20 healthy Caucasian undergraduate university students (1 male, *M*_age_ = 23; SD_age_ = 2.93) were recruited between October and December 2019 and voluntarily took part in the study. Exclusion criteria were any physiological condition of acute or chronic pain; major medical or chronic illnesses; histories of seizures, head trauma, or pregnancy; and any psychiatric or neurologic disorder. All subjects had normal-to-corrected vision and were right-handed. Participants were randomly assigned to EXP (*n* = 10) and CTR group (*n* = 10) conditions (by computer-generated randomization), and they were balanced for age (*M*_EXP_ = 23.27; SD_EXP_ = 3.64; *M*_CTR_ = 22.66; SD_CTR_ = 1.63) (Table 1). All subjects gave written informed consent before the screening phase. No payment was provided for their participation in the study. The study was carried out in accordance with the latest version of the Declaration of Helsinki and approved by the Department of Psychology of the Catholic University of the Sacred Heart of Milan, Italy.

### 2.2. Stimuli

**Figure 1:**
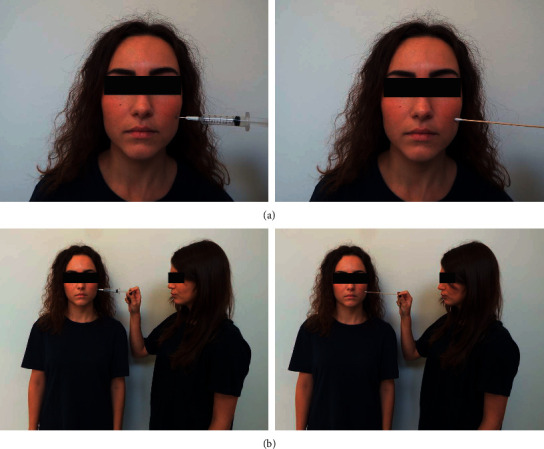
Samples of stimuli used in the study for painful (left) and nonpainful (right) stimulation in the (a) individual condition and (b) social condition.

The set of stimuli consisted of 32 pictures showing a person (male or female; opportunely randomized) receiving painful (needle penetration) or nonpainful (Q-tip touch) stimulation. The pictures also represented a single person (individual condition) or two individuals in a social interaction (social condition) for both the painful/nonpainful sets of stimuli (Figures 1(a) and 1(b)). The two individuals included in the social scenario were easily distinguishable from one another. Each picture, subtending a visual angle of 21° × 17° (width × height) at a viewing distance of 80 cm lasted 5 s and depicted individuals with neutral facial expressions. The stimuli were evaluated for the following perceptual characteristics: position, size, brightness, and content. A pool of independent judges (six Caucasian judges, 3 males, age range 20–30) controlled for gender and age evaluated the emotional neutrality of the stimuli using an adapted 5-point version of the Self-Assessment Manikin (SAM) scale [52]. Selected stimuli were rated with average values for emotional valence (*M* *=* 2.52; SD *=* 0.58) and arousal (*M* = 2.72, SD = 0.43).

### 2.3. Procedure

Subjects were seated in a dimly lit room in front of a computer monitor that was placed about 70 cm from the subject. The stimuli were presented using E-Prime 2.0 software (Psychology Software Tools Inc., Sharpsburg, PA, USA) running on a personal computer with a 15-inch screen with a visual horizontal angle of 4° and a vertical angle of 6°. A standardised set of instructions was used to explain the procedure to each participant.

Participants were required to observe each stimulus during cortical EEG oscillation recording. The participants, previously differentiated into EXP and CTR groups, were required to observe each stimulus and then asked to evaluate the observed stimuli by pressing the letter “K” for pain stimuli and “L” for no-pain stimuli on the computer keyboard. After each stimulus, participants were instructed to provide the behavioural response by pressing keyboard buttons using the right index or middle finger. The EXP group was also explicitly required to focus on its interoceptive changes while observing the stimuli and received the following instruction “During this task, we ask you to focus your attention on your bodily sensations, thoughts, and emotions. Try to observe how you feel and if there are any variations in your body as you look at the pictures,” while the CTR group received the general instruction to observe the stimuli and evaluate them for pain and no-pain. Therefore, the participants in the CTR group were not explicitly required to focus their attention on their interoceptive correlates.

Pictures were presented in a random order in the centre of a computer monitor for 5 s, with an interstimulus interval of 10 s during which participants were required to fix a central plus sign. A total of 160 stimuli were divided into 4 blocks counting 40 stimuli each. Following the disappearance of the stimulus from the monitor, during the interval participants could provide the behavioural response for painful/nonpainful stimulus features. Randomization of the order of the stimuli and blocks across participants was automatically set by E-Prime 2.0 software, to prevent potential biases due to sequence effects. At the end of the task, final manipulation checks were used, and, in this debriefing, participants were fully aware of the task and referred to the degree of attention they paid to their bodily sensations.

Potential differences in IS were assessed by applying the Heartbeat Detection (HBD) task [53] to all participants. This task was proposed after the previous task in order to avoid effects at the interoceptive level on the two groups of participants. In addition, Behavioral Inhibition/Activation System (BIS/BAS) scale [54, 55], the Interpersonal Reactivity Index (IRI) [56], and the Balanced Emotional Empathy Scale (BEES) [57] were applied to measure individual differences in personality traits, emotional, and cognitive empathy.

### 2.4. Heartbeat Detection (HBD) Task

To check the IS, all participants underwent the measure of their heartbeat perception ability by performing the mental tracking method [53], which has been proven to have good test-retest reliability [58]. Participants were seated in the same sound-attenuated room, and their heart rate was measured by applying a physiological recording tool (Biofeedback Xpert 2000, version 7.01, Schufried GmbH, Mödling, Austria). The signal was sampled at 500 Hz and analysed by a computer-based data acquisition system.

Participants were instructed to start silently counting their heartbeat when a visual start cue appeared on the PC screen (grey desktop) until they received a visual stop cue. After a brief 15 s test session, the experimental phase of the HBD task began. The experimental phase of this task consisted of 4 different time intervals of 25 s, 35 s, 45 s, and 100 s, that were all presented in random order across all participants. They were requested to type in the number of heartbeats counted at the end of each interval. During the task, participants were not allowed to take their pulse, and no feedback on the counting phase length or on their performance quality was provided. The trials were presented in random order using E-Prime 2.0 software (Psychology Software Tools Inc., Sharpsburg, PA, USA).

Interoceptive Sensitivity Index (ISI) was derived from the HBD task by calculating the mean of the four heartbeat perception intervals according to the following formula by Schandry [53]:

(1) $$\begin{array}{c} \frac{1}{4}\sum \left(1-\frac{\left[\left|\text{recorded heartbeats}-\text{counted heartbeats}\right|\right]}{\text{recorded heartbeats}}\right). \end{array}$$

According to this formula, the ISI can vary between 0 and 1, with higher scores indicating small differences between the counted and recorded heartbeats.

### 2.5. Behavioral Inhibition System and Behavioral Activation System (BIS/BAS) Measure

The BIS/BAS scale was administered for each subject by using the Italian version of Carver and White Questionnaire [54, 55] to measure individual differences in personality traits (motivational tendency to approach or avoidance behaviors. It included 24 items (20 score-items and four fillers, each measured on a 4-point Likert scale) and four scores for each subscale BIS and BAS Reward Responsiveness (BAS RR), BAS Drive (BAS D), and BAS Fun Seeking (BAS FS). The mean values and standard deviations for each scale were, respectively, for the EXP Group BIS: 27.18 (SD 5.28); BAS D: 13.45 (SD 2.01); BAS FS: 14.45 (SD 2.54); BAS RR: 21.64 (SD 1.56). For CTR group BIS: 26.11 (SD 2.80); BAS D: 13.56 (SD 2.35); BAS FS: 13.33 (SD 2.29); BAS RR: 21.44 (SD 2.35).

### 2.6. Balance Emotional Empathy Scale and Interpersonal Reactivity Index Measures

Empathy was assessed by the Italian version of the Balanced Emotional Empathy Scale by Meneghini and colleagues [57] in which participants express their level of agreement/disagreement about 30 items using a seven-point scale (from −3 to +3). In the present study, we used a seven-point scale from 1 to 7 to avoid any bias related to the attribution of negative values. Higher scores represent higher levels of emotional empathy (EXP group, *M* = 44, 73, SD = 18.58; CTR group, *M* = 28.89, SD = 14.25).

The Italian version of the interpersonal reactivity index (IRI) was used to measure both cognitive and emotional components of empathy and consists of 28-item answered on a 5-point Likert scale [56]. The measure has 4 subscales, each made up of 7 items. The fantasy scale (FS) measures the tendency to transpose oneself into fictional situations; the empathic concern scale (EC) assesses the tendency to experience compassion for unfortunate others; the perspective-taking scale (PT) measures the tendency to adopt the psychological point of view of others; the personal distress scale (PD) taps the tendency to experience discomfort in response to extreme distress in others. The mean values and standard deviations for each scale were, respectively, for the EXP group FS: 15.27 (SD 3.46); EC: 13.73 (SD 1.90); PT 16.36 (SD 2.87); PD: 13.27 (SD 3.16). For the CTR group FS: 13.89 (SD 2.36); EC: 13.44 (SD 2.12); PT 14.78 (SD 3.52); PD: 13.00 (SD 3.24).

### 2.7. EEG Recording and Reduction

EEG activity was recorded via an EEG wireless System (Live-Amp) and processed via Analyzer2 software (Brain Products GmbH, Gilching, Germany). The montage included 15 active electrodes (Fp1, Fp2, AFF3h, Fz, AFF4h, T7, C3, Cz, C4, T8, P3, Pz, P4, O1, O2; placement according to the 10–20 International System [59]. Electrode impedance was monitored for each subject prior to data collection and kept under 5 kΩ. Data were acquired using a sampling rate of 250 Hz and then filtered offline with a 0.5–45 Hz IIR bandpass filter (slope: 48 dB/octave). Data were then segmented and visually inspected for ocular, muscle, and movement artifacts. Fast-Fourier transform (Hamming window, resolution: 0.5 Hz) was applied to artifact-free segments to compute the average power spectra. Finally, the average power for the main EEG frequency bands (Delta—0.5–3.5 Hz, Theta—4–7.5 Hz, Alpha—8–12.5 Hz, and Beta—13–30 Hz) was extracted. 120-second resting baseline was registered at the beginning of the experiment before the picture series. We used this period as baseline for the successive analysis.

In the statistical analysis of the data, factors such as the lateralisation (left/right hemisphere) and the three regions of interest (ROI) grouping frontal (F: Fp1; Fp2; AFF3h; AFF4h), temporo-central (TC: T7; T8; C3; C4), and parieto-occipital (PO: P3; P4; O1; O2) electrodes were considered.

### 2.8. Statistical Methods

#### 2.8.1. Behavioral Data

*(1) Response Accuracy*. Response accuracies were calculated as the percentage of correct responses out of the total responses for pain/nonpain stimuli in the individual and social conditions. The normality of the data distribution was preliminarily assessed and confirmed by checking kurtosis and asymmetry indices. Additionally, a three-factor mixed analysis of variance (ANOVA) with *Condition* (2: individual, social) × *Pain* (2: pain, no pain) as within factors, and as between factor, the *Group* (2: EXP, CTR) was performed.

*(2) Interoceptive Sensitivity Index*. An independent-sample *t* test (IBM SPSS 25) was applied to control ISI homogeneity between groups. The normality of the data distribution was preliminarily assessed and confirmed by checking kurtosis and asymmetry indices. The threshold for statistical significance was set to *α* = 0.05. Equality of variances between groups was checked by Levene's test which was computed to test homogeneity of variances between the two groups and to adapt the computation of subsequent inferential tests accordingly.

*(3) BIS/BAS, BEES, and IRI*. An independent-sample *t* test was applied to all BIS/BAS, BEES, and IRI scores to control individual differences in personality and empathy traits between groups. The normality of the data distribution was preliminarily assessed and confirmed by checking kurtosis and asymmetry indices. The threshold for statistical significance was set to *α* = 0.05. Equality of variances between groups was checked by Levene's test was computed to test homogeneity of variances between the two groups and to adapt the computation of subsequent inferential tests accordingly.

#### 2.8.2. Electrophysiological (EEG) Data

Four mixed repeated measures ANOVA with independent within factors *Condition* (2: individual, social stimuli) × *Pain* (2: pain, no pain) × *Lateralisation* (2: left, right) × *Region of Interest* (ROI) (3: frontal, temporo-central, and parieto-occipital), and as between factor the *Group* (2: EXP vs CTR) was applied to dependent EEG data (power data). This mixed repeated measures ANOVA was performed for each frequency band (Delta, Theta, Alpha, and Beta) to highlight the differences between the two groups. Pairwise comparisons were applied to the data in case of significant effects. Simple effects for significant interactions were further checked via pairwise comparisons, and Bonferroni correction was used to reduce multiple comparisons' potential biases. For all the ANOVA tests, the degrees of freedom have been corrected using Greenhouse–Geisser epsilon where appropriate. Furthermore, the normality of the data distribution was preliminarily assessed and confirmed by checking kurtosis and asymmetry indices. The size of statistically significant effects has been estimated by computing partial eta squared (*η*^2^) indices. Brain Vision Analyzer 2.0 (Brain Products GmbH, Munich, Germany) was used for EEG data visualization.

## 3. Results

**Figure 2:**
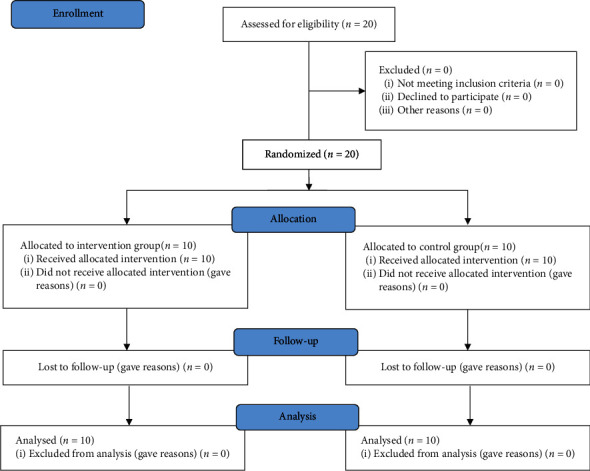
CONSORT flow chart.

All participants (20 subjects, divided equally by EXP and CTR group) were included in the analysis and results; no participant was excluded (Figure 2).

### 3.1. Behavioural Results

Response accuracy for pain/nonpain stimuli in the individual and social conditions was high for both groups. During the pain judgment in the individual condition, the accuracy for painful stimuli was 100% for the EXP group and 99% for the CTR group, while the accuracy for the nonpainful stimuli was 100% for both the EXP and the CTR groups. In the social condition, the accuracy for painful stimuli was 100% for the EXP group and 93% for the CTR group, while the accuracy for the nonpainful stimuli was 99% for the EXP group and 100% for the CTR group. ANOVA revealed no significant difference in response accuracy between groups (all *p* > 0.05).

*Interoceptive Sensitivity Index.* No significant differences were found in the ISI measure between the two groups (*M*_*EXP*_ = 0.51; SD_EXP_ = 0.21; *M*_CTR_ = 0.62; SD_CTR_ = 0.15) (all *p* > 0.05).

*BIS/BAS, BEES, and IRI.* No significant differences were found in BIS/BAS, BEES, and IRI scores between the two groups (all *p* > 0.05).

### 3.2. EEG Task-Related Electrophysiological Results

#### 3.2.1. Delta Band

**Figure 3:**
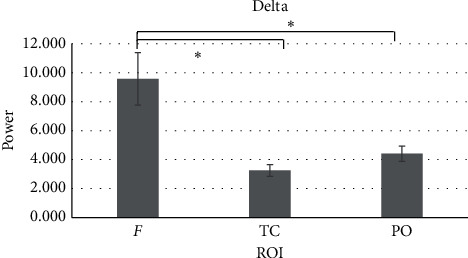
Delta band. The bar chart shows the higher presence of delta power values in frontal brain regions for both groups. In the chart, bars represent ±1 SE; all asterisks mark statistically significant differences, with *p* ≤ 0.05. *Abbreviations.* ROI: region of interest; F: frontal; TC: temporo-central; PO: parieto-occipital.

For delta band, a main effect for ROI was found (*F*_[1.093,18]_ = 11.099, *p* ≤ 0.001, *η*^2^ = 0.381). Pairwise comparisons revealed significant higher mean values for *F* compared to TC (*F*_[1,17]_ = 6.439, *p*=0.007*, η*^2^ = 0.431) and PO (*F*_[1,17]_ = 6.439, *p*=0.017, *η*^2^ = 0.431) (Figure 3). No other significant effects were found.

#### 3.2.2. Theta Band

About theta, a main effect for ROI was found (*F*_[1.264,18]_ = 4.509, *p*=0.018, *η*^2^ = 0.200). Pairwise comparison revealed a significant increased activity in PO compared to TC areas (*F*_[1,17]_ = 8.959, *p*=0.003, *η*^2^ = 0.513).

Moreover, an interaction effect group × condition ×pain × lateralisation × ROI was found (*F*_[2,18]_ = 3.639, *p*=0.036, *η*^2^ = 0.168).

**Figure 4:**
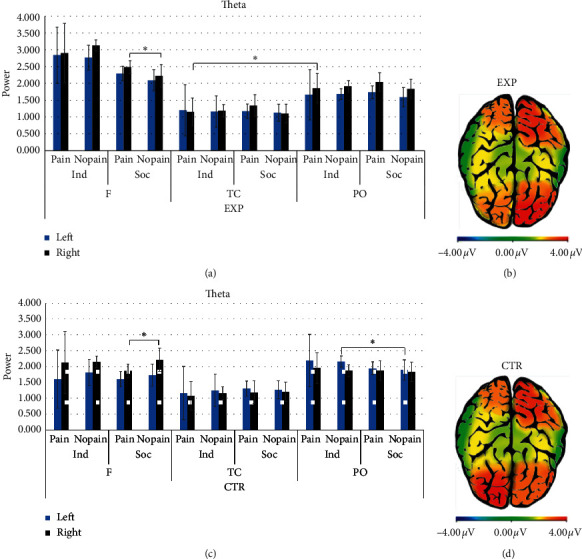
Theta band. (a) Bar charts show theta power mean values in the EXP group. (b) Theta power representation for the EXP group. The red area represents the increase of theta power in the right hemisphere for painful stimuli, with a frontal activation for the social condition (left head) and a parieto-occipital increase for the individual condition (right head). (c) Bar charts show theta power mean values in the CTR group. (d) Theta power representation for the CTR group. The red area represents the increase of theta power for nonpainful stimuli, with a right frontal activation for the social condition (left head) and a left parieto-occipital activation for the individual condition (right head). For all charts, bars represent ±1 SE; all asterisks mark statistically significant differences, with *p* ≤ 0.05. *Abbreviations*. EXP: experimental group; CTR: control group; F: frontal; TC: temporo-central; PO: parieto-occipital; Ind: individual condition; Soc: social condition; pain: painful stimuli; no pain: nonpainful stimuli.

Pairwise comparisons revealed for the EXP group, firstly, in the right frontal ROI a significant higher activation for painful compared to nonpainful stimuli in the social condition (*F*_[1,17]_ = 4.727, *p*=0.043, *η*^2^ = 0.208); secondly, a significant higher activation of PO compared to TC areas in the right hemisphere for the painful stimuli in the individual condition (*F*_[1,17]_ = 6.336, *p*=0.023, *η*^2^ = 0.427) (Figures 4(a) and 4(b)).

For the CTR group, pairwise comparisons revealed firstly a significant higher activation in left PO regions for nonpainful stimuli in the individual compared to social condition (*F*_[1,17]_ = 6.634, *p*=0.036, *η*^2^ = 0.002); secondly, in the right frontal ROI, a significant higher activation for nonpainful compared to painful stimuli in the social condition (*F*_[1,17]_ = 6.634, *p*=0.019, *η*^2^ = 0.269). No other significant effects were found (Figures 4(c) and 4(d)).

#### 3.2.3. Alpha Band

**Figure 5:**
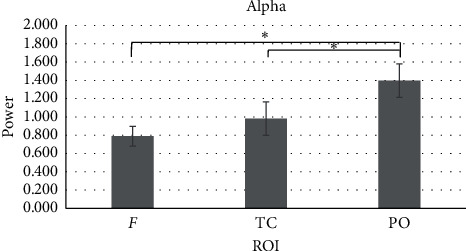
Alpha band. The bar graph shows the higher presence of alpha power values in parieto-occipital brain regions for both groups. In the chart, bars represent ±1 SE; all asterisks mark statistically significant differences, with *p* ≤ 0.05. *Abbreviations.* ROI: region of interest; F: frontal; TC: temporo-central; PO: parieto-occipital.

Regarding alpha band, a main effect for ROI was found (*F*_[2,18]_ = 14.860, *p* ≤ 0.001, *η*^2^ = 0.452). Significant higher presence of alpha power (decreased activity) was detected in PO compared to F (*F*_[1,17]_ = 14.191, *p* ≤ 0.001, *η*^2^ = .625) and TC regions (*F*_[1,17]_ = 14.191, *p*=0.003, *η*^2^ = 0.625) (Figure 5). No other significant effects were found.

#### 3.2.4. Beta Band

For beta band, a main effect for ROI was found (*F*_[2,18]_ = 3.886, *p*=0.030, *η*^2^ = .178). Pairwise comparisons revealed significantly higher beta values in PO compared to TC (*F*_[1,17]_ = 6.319, *p* ≤ 0.005, *η*^2^ = 0.426).

**Figure 6:**
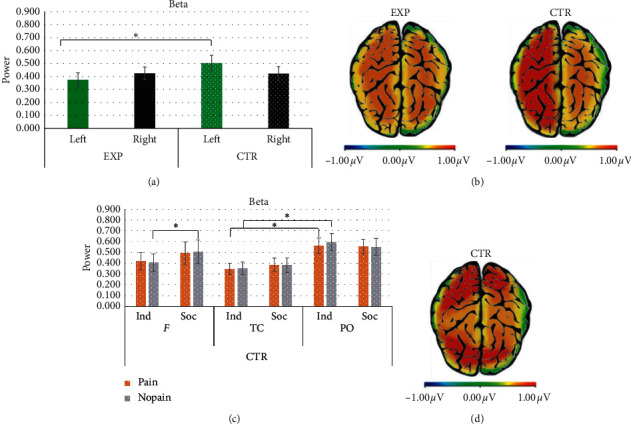
Beta band. (a) Bar charts show beta power lateralisation in the two groups. (b) Beta power representation for the EXP and the CTR group. The red area represents the increase of beta power mean values in the left hemisphere for CTR compared to EXP. (c) Bar charts show beta power mean values in the CTR group. (d) Beta power representation for the CTR group. The red area represents the increase of beta power in frontal areas for nonpainful stimuli in the social condition (left head), and in parieto-occipital areas for nonpainful stimuli in the individual condition (right head). For all charts, bars represent ±1 SE; all asterisks mark statistically significant differences, with *p* ≤ 0.05. *Abbreviations.* EXP: experimental group; CTR: control group; F: frontal; TC: temporo-central; PO: parieto-occipital; Ind: individual condition; Soc: social condition; pain: painful stimuli; no pain: nonpainful stimuli.

Secondly, a significant interaction group × lateralisation effect was found (*F*_[2,18]_ = 4.547, *p*=0.047, *η*^2^ = 0.202). Pairwise comparison revealed higher beta values in the left hemisphere for the CTR compared to EXP group (*F*_[2,18]_ = 2.590, *p* ≤ 0.001, *η*^2^ = 0.126) (Figures 6(a) and 6(b)).

Thirdly, an interaction effect group × condition × pain × ROI was found (*F*_[2,18]_ = 3.378, *p*=0.045, *η*^2^ = 0.158). For the CTR group, pairwise comparison revealed, in frontal ROI, a significant higher presence of beta power for nonpainful stimuli in the social compared to the individual condition (*F*_[1,17]_ = 4.847, *p*=0.041, *η*^2^ = 0.212). In addition, pairwise comparison showed that, in the individual condition, PO was found to be more responsive with respect to TC, both for painful (*F*_[1,17]_ = 5.013, *p*=0.013, *η*^2^ = 0.371) and nonpainful stimuli (*F*_[1,17]_ = 5.487, *p*=0.010, *η*^2^ = 0.392) (Figures 6(c) and 6(d)).

## 4. Discussion

The present study provided new insights into the way interoception may affect the representations of observing others in painful conditions, based on EEG measurements. Indeed, the modulation of IA was previously supposed to influence neural correlates of observation of pain in others. However, this aspect has not been tested with neurophysiological protocols so far. Therefore, we expected that the EXP group would display enhanced emotional and cognitive responsiveness to the observation of pain in others compared to the CTR group.

The following main results were obtained in relation to the power of the frequency bands and will be discussed below point by point. Firstly, a hemispheric lateralisation effect was found, with right lateralisation of the theta band for the EXP group when observing painful stimuli. Secondly, an enhanced left activation of the theta and beta bands for the CTR group with respect to the EXP group was found, particularly when observing nonpainful stimuli. Thirdly, the role of the frontal regions in social cognition processing was confirmed to be sensitive to stimuli presented in the social condition, while parieto-occipital electrodes responded to the individual condition for both groups.

Starting with the first evidence of this study, for the EXP group, significantly higher theta power was found in the following two areas of the right hemisphere in response to painful stimuli: (i) in the frontal area for the stimuli presented in the social condition and (ii) in the parieto-occipital area for stimuli displayed in the individual condition. The theta band underlies the mechanisms for cognitive control over the situation [60], alertness, attention, and readiness to process the presence of emotional information [44–50], activation of memory, and emotion regulation systems [51]. In addition, the theta duration was interpreted as a correlate of increased attention and arousal due to the emotional content of the stimulus [48]. Moreover, regarding the electrodes where these effects occur, it is worth noting that the modulation of the theta band by prefrontal and frontal cortex regions (DLPFC) has been previously shown to be related to cognitive control over salient emotional stimuli [47] and to the affective and motivational evaluation of pain in others [29–31]. Instead, the theta band over parieto-occipital areas was detected in response to the visual aspects of the stimuli, to the observation (rather than the direct stimulation and induction) of a painful condition or to the arousing levels of the emotional visual stimuli presented [61]. In the context of the observation of pain in others, previous evidence has shown an increase in the theta band in frontal areas elicited by painful stimuli and higher subjective ratings of perceived pain and self-unpleasantness, thus suggesting a role of this band in the affective processing of empathy for pain [15].

Therefore, it is possible to suppose that the increase of the theta band in the frontal regions for the EXP group could be considered as a marker of the amplification of emotional response mainly in relation to painful unpleasant stimuli. Considering that this group was explicitly required to direct the attention on its interoceptive states, it might be that the higher theta power in parieto-occipital electrodes (posterior parietal and occipital cortex) compared to temporo-central areas could suggest that the associative elaboration processing, in addition to the emotional one, is mainly conveyed to painful stimuli in this group.

In more detail, for the EXP group, theta oscillation in relation to painful stimuli was detected in the right hemisphere. This outcome could be better explained by considering the interpretative models that linked the hemispheric lateralisation response to the emotional stimuli valence. In fact, the presence of the theta band as a marker of emotional processing in the right hemisphere could be supported by the right hemisphere hypothesis (RHH) [62, 63], according to which the right hemisphere is responsible for the elaboration of emotional content. In addition, more recently, the valence-specific hypothesis (VSH) [48, 50, 64–66] argued that both hemispheres process emotion (not only the right side) and that each hemisphere is specialised for valence-specific emotion, with the left hemisphere more dominant for positive emotions and the right hemisphere more dominant for negative emotions [65, 67].

In line with this last model, it is possible to suggest that the IA in the EXP group first enhanced the emotional responsiveness towards painful stimuli, reflected by theta band activation, and that this emotional representation has a negative valence, as suggested by the right hemisphere involvement. Previously, an interrelationship between right anterior insula activation, interoceptive accuracy, and subjective negative emotional experience was found [6]. Moreover, enhanced attention to interoceptive states is the characteristic of anxiety disorders [68]. This evidence can help to support the link between the interoceptive experience and the valence of the emotional response for the EXP group, showing a negative emotional representation for painful stimuli.

Secondly, the absence of an explicit interoceptive focus in the CTR group might have led to a left hemispheric response and heightened attention toward nonpainful stimuli. Indeed, for the CTR group, the theta band increased for nonpainful stimuli in the two following areas: (i) in the right frontal area for stimuli in the social condition and (ii) in the left parieto-occipital site for stimuli displayed in the individual condition. Hence, for the CTR group, for which a deep analysis of one's own interoceptive experience related to the situation was not prescribed, the emotional amplification suggested by the theta presence in the frontal area was mainly evoked by nonpainful stimuli. In line with this, the associative processing reflected by theta in the parieto-occipital area could perhaps indicate that their attention was mainly channelled towards nonpainful cues. The theta band in left parieto-occipital sites was previously found during the observation of pain in others [34], and in the case of the CTR group, perhaps this might imply the salience of nonpainful rather than painful stimuli. However, in a study by Mu and colleagues [15], the modulation of theta was explored mainly in response to painful and neutral stimuli but not to nonpainful stimuli. Therefore, to date, there has not been a univocal explanation for the manifestation of theta when observing nonpainful stimuli.

According to the VSH, we can argue that a left hemispheric lateralisation might reflect a more positive attitude towards a cue where painful stimulation is absent, and a positive response could be ordinary and plausible. Nevertheless, the right frontal activation for nonpainful stimuli detected for this group could be in contrast to that described with the VSH. This last effect could be more in line with the RHH and makes these interpretations lean towards a predominant emotional processing of nonpainful stimuli, regardless of valence, in the CTR group.

Concerning the beta band, for the CTR group, a significantly higher presence of beta power was found (i) in the frontal regions for nonpainful stimuli displayed in the social condition and (ii) in parieto-occipital electrodes for both painful and nonpainful stimuli in the individual condition compared to temporo-central electrodes. Besides, significant general responsiveness of the left hemisphere was found for the CTR group compared to the EXP group. This lateralisation effect could still be explained by the previously mentioned model exploring hemispheric left/right frequency bands analysis in the context of emotions, namely, the VSH. Hence, major left hemispheric responsiveness could suggest positive engagement in controls that were more cognitively engaged in the observation of actions than in observing one's feelings. However, previous studies on emotion have mainly shown left-lateralised modulation of alpha [64, 65, 69], delta, and theta low-frequency bands in response to stimuli with positive valence [47, 49, 50]. Accordingly, future studies are needed to unveil this effect of the left beta band at the cognitive representational level during the observation of pain in others.

While from a broad perspective, beta band activity has been related to the maintenance of the current sensorimotor or cognitive state [70], and other studies have suggested its relationship with attentional processes and visual awareness [71]. Specifically, the frontal beta activity might indicate, at a cognitive level, maintenance of the cognitive state, top-down endogenous elaboration processing, and successful inhibition of the motor response [70, 72], while its presence over parieto-occipital sites suggests ongoing visual attention processes [71]. In this case, a possible explanation could be that the presence of beta in the CTR group indicates the ongoing top-down cognitive processing towards nonpainful stimuli when is detected in frontal regions, while its parietal manifestation could involve basic visual attentional processing towards painful and nonpainful stimuli.

In the context of the observation of pain in others, the absence or suppression of the beta band over central brain areas goes hand in hand with the emotional and cognitive features of the phenomenon, which relegates the role of the beta band as a marker of the sensory qualities of pain, even when experienced vicariously [19, 42]. Nonetheless, in this case, it is possible to suppose that the modulation of beta for the CTR group, which was exposed to the observation of pain in others but did not explicitly focus on its interoceptive correlates, could be considered a marker of top-down cognitive appraisal of the situation.

Since the cortical generators of EEG oscillations determine their functional meaning related to cognitive and emotional processing, these reflections must also be extended to the brain regions in which these cortical oscillations occurred and were detected. However, given the low spatial resolutions of the EEG signal, the following considerations need to be taken with caution and future research could integrate and confirm this evidence with neuroimaging techniques, such as magnetoencephalography. Interesting effects were mainly found to involve a fronto-parietal network, which was previously found to be specialised for representing one's own and others' responses to painful stimuli [23, 24] and for top-down elaboration processing of pain [73]. The integrated activity of a high-order network starting from the conscious appraisal of the eliciting visual stimulus (temporo-parietal areas) to the encoding of internal states and short-term memory (prefrontal and dorsolateral prefrontal areas) supports high-order phenomena for which observing other people's pain might modulate our own representations [74]. The interpretations outlined in this study should be taken cautiously because, so far, an overarching framework for the significance of cortical oscillations for pain is still lacking, and previous authors have mapped the modulation of theta and beta presence in patients with chronic pain experiences [40].

Thirdly, as a common thread running through the results found for theta and beta bands, both for the EXP group and the CTR group, the stimuli presented in the social condition engaged the frontal sites more than the individual condition. Indeed, for both groups, a predominant right frontal manifestation of the theta band was found for painful/nonpainful stimuli displayed in the social compared to the individual condition. The social scenario might have activated the greater emotional reaction for both groups. Higher-order social functions, such as an empathic response to pain, were previously shown to elicit increased brain responses within the PFC (specifically the DLPFC) [74, 75]. Increased PFC activation and skin conductance response were found when subjects empathised with the interpersonal scenario [76, 77], together with a hemispheric lateralisation effect according to the valence of the interpersonal stimuli (left for positive and right for negative) [78].

These previous studies did not manipulate the participants' IA, which is linked to their neural and psychophysiological implicit response, and they did not test if manipulation of IA may have an impact on their body-brain response features. So far, few studies have explored the observation of pain in others using stimuli consisting of dual interpersonal scenarios. Nonetheless, understanding pain in others in a social dynamic and our evidence suggest the social context in which stimuli are presented may have an impact on emotional and cognitive neurophysiological correlates.

This could be due to activation of the frontal part of the mirror-neuron system found during the perception of a painful social situation intentionally inflicted by another individual [79]. In contrast, enhanced theta and beta PO responsiveness was found for both groups observing stimuli, regardless of pain connotation, in the individual condition. One possible explanation could be that this condition might have activated a temporo-parietal-occipital network involved in self-processing and individual mirror experiences [80] and motor mirroring [81].

Here, the adoption of the pain framework was chosen as a test bench to demonstrate that these effects were not a generic result of interoception but a specific outcome that mirrors the representation of observing pain in others. Indeed, the observation of the pain of others takes on primarily emotional, rather than sensory, nuances, in particular, if individuals focus on one's interoceptive correlates. To draw individuals' attention towards how they feel might, therefore, accentuate the perception of negative emotional states associated with the painful condition in others (as shown by a right-side hemispheric effect), while in the CTR group, which did not explicitly focus the attention on its interoceptive correlates, this effect was lacking or at least softened. On the other hand, for this group, cortical responsiveness for nonpainful stimuli emerged, with prominent left lateralisation previously found for stimuli with positive and pleasant valence. Going down with the specifics and observing significant analytic differences, the social context in which the stimuli were presented was not a key point for the effects of IA on the observation of pain. Nonetheless, an effect for the individual and social stimuli conditions was found for both groups. The interest in exploring individual and social conditions came from recent social neuroscience paradigms suggesting the highest ecological validity of stimuli depicting interpersonal interactions. Future research could go in this direction to deeply analyse the systematic effects of individual/social polarity.

Moreover, in the present research, no significant group effects for alpha and delta bands were observed, as was shown in previous research on the observation of pain in others and on emotional behaviour. These different results may be due to the heterogeneous methodologies used, the specific modulation of IA, and/or the time range of the epoch considered for this study, which was greater than those adopted in the previous research (1000 ms).

The present preliminary research was needed to explore the effects that the focused attention on ongoing interoceptive states can have on a salient emotional and cognitive condition, such as pain. In the EXP group, we observed that IA was able to widen the salience and negative valence of painful conditions. Previous findings have suggested that the more aware a person is of ongoing bodily processes, the more successful this person's emotional regulation in response to a negative affect will be [82]. Accordingly, we suppose IA could be modulated to promote emotion regulation and an empathic response.

In the end, despite the innovativeness of this work, some limitations and future suggestions for improved research practices should be considered. Firstly, the integration of other neuroscientific tools and techniques, such as functional near-infrared spectroscopy (fNIRS), autonomic indices, and evoked related potentials (ERPs), could be used to investigate the hemodynamic and peripheral correlates underlying interoceptive experience and the time-related different emotional states and mechanisms characterising the observation of pain in others. fNIRS was recently demonstrated to be informative for exploring cortical hemodynamic response in empathy for pain studies [83]. Moreover, given the low spatial resolutions of the EEG signal, the considerations derived from ROIs need to be taken with caution and future research could overcome this limitation by designing a fNIRS-EEG co-registration study. In addition, the role of the vMPFC (a key region responsible for dealing with affective feelings) and insula (responsible for processing bodily sensations) should be explored by neuroimaging future studies. Secondly, given the exploratory nature of this study, no sample size calculation using power was adopted; therefore, the sample size should be increased and also balanced for gender, to confirm and generalise the present evidence. Indeed, our sample was relatively small and mainly composed of females; therefore, its generalisability and external validity are of medium level and can be increased; future studies should include male population (only or balanced homogenously with females) to examine and exclude possible gender effects. Thirdly, the intersubjective differences related to vicarious pain experiences (sensory localised versus affective nonlocalised pain responders) and some personality components (such as empathy as a trait; [84, 85]) should be explored as potential stable components that could explain neurophysiological differences. Indeed, possible interindividual features could have modulated the central responses based on subjective empathic and emotional responsiveness to painful/nonpainful situations. Therefore, future research may consider (i) the application of correlation and regression analyses between individual differences (IS, personality and empathic traits) and EEG data, (ii) the development of a double-blind randomized controlled trial (in fact, participants received explicit instructions from the investigator on the interoceptive task, while the link between pain recognition and the interoceptive attentiveness was not revealed), and (iii) to replicate this study on larger samples to deepen the significant between-group differences, strengthening the empirical observations and related interpretations.

## 5. Conclusions

To conclude, the present study highlighted how the modulation of IA could lead to an enhancement of specific cortical effects related to the emotional and cognitive representations of observing pain in others. Specifically,

- Most informative significant results of the interoception modulation in the context of observation of pain in others were found in relation with theta and beta cortical oscillations.
- The group that was explicitly directing the attention toward the interoceptive correlates displayed a negative echo while observing painful stimuli in social and individual conditions. Therefore, IA might enhance the emotional representation of painful stimuli, highlighting their negative and unpleasant features, as reflected by the theta band in the right hemisphere.
- While the attention of the control group was mainly on nonpainful stimuli in social and individual conditions, suggesting an ongoing top-down cognitive processing of these stimuli.
- Also, in both groups, frontal cortical regions were found to be more significantly sensitive to scenarios depicting social interactions. This could be due to the social-cognitive demand nature of social stimuli, inducing emotional mirroring and requiring deeper analysis of the social context, perceived intentionality, and interpersonal dynamic. In contrast, the posterior parietal activation found for stimuli presented in the individual condition could be addressed with regards to motor mirroring or self-processing.

Overall, frequency band analysis allowed us to obtain information on how the EEG cortical oscillations signaled the induction of an explicit interoceptive experience and its emotional and cognitive representation, during the observation of pain in others.

## Figures and Tables

**Table 1:** Participants' demographic data.

Variable	Group EXP (*n* = 10)	Group CTR (*n* = 10)
Age (Y)	23.27 ± 3.64	22.66 ± 1.63
Sex (male/female)	(1/9)	(0/10)

Data are presented as the means ± SD or numbers of participants. Group CTR: control group; group EXP: experimental group. No significant differences were observed between the groups.

## Data Availability

The datasets used and/or analysed during the current study are available from the corresponding author on reasonable request.
